# A quantitative spatiotemporal analysis of microglia morphology during ischemic stroke and reperfusion

**DOI:** 10.1186/1742-2094-10-4

**Published:** 2013-01-11

**Authors:** Helena W Morrison, Jessica A Filosa

**Affiliations:** 1 Georgia Health Sciences University, 1120 15th, St, Augusta, GA, 30912, USA

**Keywords:** Microglia, Morphology, Mouse, Ischemic stroke, Reperfusion

## Abstract

**Background:**

Microglia cells continuously survey the healthy brain in a ramified morphology and, in response to injury, undergo progressive morphological and functional changes that encompass microglia activation. Although ideally positioned for immediate response to ischemic stroke (IS) and reperfusion, their progressive morphological transformation into activated cells has not been quantified. In addition, it is not well understood if diverse microglia morphologies correlate to diverse microglia functions. As such, the dichotomous nature of these cells continues to confound our understanding of microglia-mediated injury after IS and reperfusion. The purpose of this study was to quantitatively characterize the spatiotemporal pattern of microglia morphology during the evolution of cerebral injury after IS and reperfusion.

**Methods:**

Male C57Bl/6 mice were subjected to focal cerebral ischemia and periods of reperfusion (0, 8 and 24 h). The microglia process length/cell and number of endpoints/cell was quantified from immunofluorescent confocal images of brain regions using a skeleton analysis method developed for this study. Live cell morphology and process activity were measured from movies acquired in acute brain slices from GFP-CX3CR1 transgenic mice after IS and 24-h reperfusion. Regional CD11b and iNOS expressions were measured from confocal images and Western blot, respectively, to assess microglia proinflammatory function.

**Results:**

Quantitative analysis reveals a significant spatiotemporal relationship between microglia morphology and evolving cerebral injury in the ipsilateral hemisphere after IS and reperfusion. Microglia were both hyper- and de-ramified in striatal and cortical brain regions (respectively) after 60 min of focal cerebral ischemia. However, a de-ramified morphology was prominent when ischemia was coupled to reperfusion. Live microglia were de-ramified, and, in addition, process activity was severely blunted proximal to the necrotic core after IS and 24 h of reperfusion. CD11b expression, but not iNOS expression, was increased in regions of hyper- and de-ramified microglia during the course of ischemic stroke and 24 h of reperfusion.

**Conclusions:**

Our findings illustrate that microglia activation after stroke includes both increased and decreased cell ramification. Importantly, quantitative analyses of microglial morphology and activity are feasible and, in future studies, would assist in the comprehensive identification and stratification of their dichotomous contribution toward cerebral injury and recovery during IS and reperfusion.

## Background

Ischemic stroke is a leading cause of mortality, morbidity and disability in the US [1]. An absence of oxygen and nutrients precipitates an abundance of deleterious events such as neuronal depolarization and cytotoxic edema, among others, to cause immediate neurological dysfunction, and results in an irreversible necrotic core [2]. The extension of the necrotic core into the penumbra is influenced by additional factors such as regional differences in the composition of brain tissue, the vulnerability of different cell types to ischemia, residual tissue perfusion and additional events such as reperfusion [2-4]. In addition, it is generally accepted that both systemic (neutrophils and monocytes) and local (microglia) immune cells contribute, as individual cell populations and/or in concert, toward the extension of the necrotic core after ischemic stroke and reperfusion through proinflammatory mechanisms [2,5-8]. However, the inflammatory response responsible for phagocytosis and preparation for wound healing may also promote brain recovery after ischemic stroke and reperfusion [7,9]. This complex and confounding scenario has yet to be fully elucidated, limiting the development of novel stroke therapies.

Microglia, as the resident brain immune cells, have a surveillance function characterized by the continuous monitoring of their surrounding microenvironment in a ramified morphology [10,11]. In general, ramified cells have small somas and an extensive arborization of dynamic processes, necessary for active surveillance of microglia microdomains. However, the ramified morphology and process activity vary across brain regions [12]. Microglia’s arborized appearance distinguishes them from infiltrating systemic immune cells; consequently, cell morphology plays an important role in differentiating between local and systemic immune responses. Microglia are consistently distributed throughout brain parenchyma leaving few areas, if any, without constant surveillance; however, cell density between brain regions is variable [5,7,13].

Microglia are particularly well positioned for an immediate response to deleterious ischemic and reperfusion events. Although ramified in the healthy brain, microglia morphology is varied during transformation, upon “activation,” to a phagocytic phenotype [7]. Many describe this morphological change as a de-ramification in which the number of processes and process length are progressively attenuated until the cell displays an amoeboid morphology [14-18]. As such, microglia ramification is an objective measure of microglia responses after ischemic stroke and reperfusion. Microglia process motility, highly active in the healthy brain, is progressively attenuated in a spatial relationship to acute physical or chemical injury [10,11,19]. Importantly, microglia de-ramification and attenuated process motility are a correlative response upon microglia activation [14].

Microglia are considered constitutively active cells because of their continuous surveillance function. Altered microglia function, beyond surveillance, is likely the result of disrupted brain cell function and/or an altered balance of inhibition and activation input [17]. In addition to a variable morphology and motility, microglia are pleiotropic when stimulated to respond beyond a surveillance function. These pleiotropic functions may be identified through an increased expression of cell-surface receptors and release of cytokines and chemokines, which may result in proinflammatory and neurotrophic actions [18]. Correlating microglia form to function is difficult as there are few tools to adequately quantify morphological changes. Despite these limitations, recent research suggests that the ramified morphology is purposeful beyond tissue surveillance and may include process-directed phagocytosis [20] and neurotransmitter modulation [21], whereas the amoeboid morphology, observed after severe or extended injury, is an indicator of full transformation toward the macrophage phenotype. While changes in morphology may associate with alterations in microglia action, microglia function may also change independent of morphology [7]. Taken together, microglia’s dynamic pleomorphic and pleiotropic nature confounds our understanding of microglia’s role as a participant in neurodegenerative or neuroprotective actions during the evolution of cerebral injury after ischemic stroke and reperfusion.

Quantitative methods to assess microglial ramification in fixed tissue and live cell imaging are limited, an obstacle in precisely characterizing microglial morphological responses to injury. For this reason, our objective was to examine the spatiotemporal progression of microglia morphology related to the evolution of cerebral injury induced by ischemic stroke and reperfusion. We hypothesized that ischemia and reperfusion would elicit differing microglia morphological responses and that a spatiotemporal relationship exists between microglia morphology and evolving brain injury after ischemic stroke and reperfusion. To test these hypotheses, we developed a novel method to quantitatively analyze microglia morphology in a murine model of transient focal ischemic stroke.

## Methods

### Animals

C57Bl/6 mice (Jackson Laboratories, Bar Harbor, ME, no. 00064, 20–25 g, male) were used for all *in vitro* experiments to quantify microglia morphology after ischemic stroke and reperfusion. Male CX_3_CR_1_-GFP-targeted mice (Jackson Laboratories, no. 005582) were bred to female C57Bl/6 mice (background to the CX_3_CR_1_-GFP). The heterozygous offspring (GFP^−/+^, 20–25 g, male) were used for *ex vivo* experiments to quantify the morphology of live microglia after ischemic stroke and 24-h reperfusion. All animal experiments were performed in compliance with the Georgia Health Sciences University Institutional Animal Care and Use Committee.

### Temporary middle cerebral artery occlusion

A filament method was used to induce a temporary middle cerebral artery occlusion (tMCAO) as previously described [22]. Briefly, mice were anesthetized with 1–2% isoflurane throughout the procedure in a medical air/oxygen mixture (0.4 l/min and 0.1 l/min, respectively). Focal ischemia was induced by temporary occlusion of the right common carotid artery and filament advancement, via the internal common carotid artery, to the ostea of the middle cerebral artery. The ischemic period for all experiments was 60 min, continuously verified by laser Doppler measures of relative cerebral blood flow to the MCA territory (Perimed Periflux 5000, North Royalton, OH). Animals were included in the study if cerebral ischemia (filament placement and temporary carotid ligation) resulted in at least a 70% reduction of blood flow and reperfusion (filament removal and carotid release) resulted in at least a 70% return of blood flow when compared to the animal’s baseline blood flow. The sham procedure included all elements up to filament placement (for the 24-h reperfusion group only, *n* = 6). Deviations from procedures in this study as compared to previously published procedures only pertain to the duration of reperfusion (ischemia only, *n* = 10; 8-h reperfusion, *n* = 7; 24-h reperfusion, *n* = 10) prior to animal sacrifice and data collection. Of the mice entered into the study, all were included, and no mice died prior to data acquisition.

### Immunohistochemistry

Brain samples were removed from euthanized animals at three time points (ischemia, ischemia with 8 and 24 h of reperfusion). Whole brains were fixed for 24 h in 4% paraformaldehyde followed by 72 h incubation in a 30% sucrose solution. Brains were stored at −80°C until sectioning. Propidium iodide (1.5 mM, Sigma, P4170) was injected into the circulation by cardiac puncture just prior to animal euthanasia and brain removal to assess for the presence of cell death induced by ischemia and 24 h reperfusion. Propidium iodide quickly diffuses to the cell nucleus of necrotic cells and, when bound to nucleotides, fluoresces a bright red [23]. Frozen whole brains were cut into coronal sections (Leica cryostat CM3050, 200 μm) and stored frozen in a cryoprotectant solution (50% 50 mM PBS, 30% ethylene glycol, 20% glycerol) until tissue processing for immunohistochemical staining. To identify microglia and neurons, free-floating brain sections were blocked in 10% horse serum solution (0.01M PBS 0.05% Triton X 0.04% NaN_3_) for 1 h followed by a 24-h incubation with primary antibodies (rabbit anti-Iba-1 1:1,000, Wako no. 019–19741; mouse anti-NeuN 1:1,000, Millipore MAB377). A 4-h secondary incubation followed (donkey anti-rabbit-Alexa 488 no. 711-546-152, 1:250, donkey anti-mouse-Dylight 649 no. 715-606-150, 1:250; Jackson ImmunoResearch Laboratories). In an independent cohort, sections were blocked as described and incubated for 72 h with rat anti-CD11b/CD18 primary antibody (1:100, Chemicon International MAB1387Z) and rabbit anti-Iba-1 followed by a 4-h secondary incubation (donkey anti rat-CY3 no. 712-166-153, 1:250; donkey anti-rabbit-Alexa 488, 1:250; Jackson ImmunoResearch Laboratories). All reactions occurred at room temperature and were washed between incubations with 0.01M PBS for 15 min. Slices were washed and mounted to slides using Vectashield (Vector Laboratories, H-1000).

### Image acquisition and skeleton analysis

**Figure 1:**
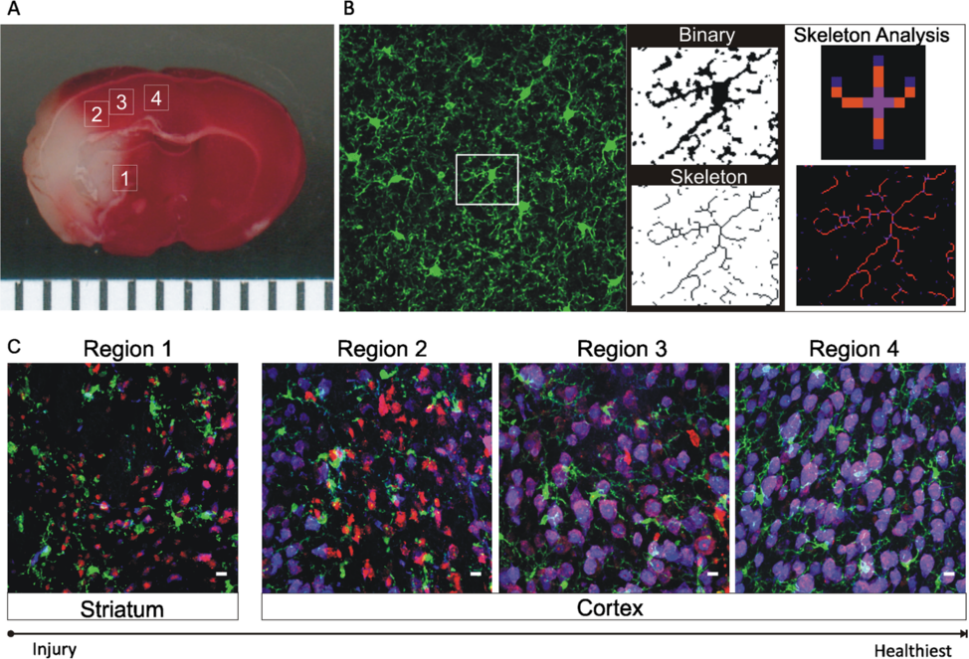
**Skeleton analysis of microglia morphology. A** Confocal images were acquired in brain regions 1–4 (1 striatum; 2–4 cortex) as illustrated in the TTC image of a typical area of injury after ischemic stroke and 24 h reperfusion (white tissue is necrotic, and red tissue is healthy). Brain regions were conserved across time points in the ipsilateral, contralateral and sham hemispheres. **B** Maximum intensity projections of confocal images were converted to binary images and then skeletonized. The number of microglia process endpoints (blue) and process length (orange) were summarized for statistical comparisons from Analyze Skeleton plugin by Image J. **C** Composite images of neurons, microglia and cell death from ipsilateral brain regions 1–4 [blue-neuron (anti-NeuN), green-microglia (anti-iba-1), red-cell death (propidium iodide)]. Scale bar = 10 μm.

A skeleton analysis method was developed to quantify microglia morphology in immunofluorescent images of fixed brain tissue. Confocal images (21-μm z-stack at 3-μm intervals, Zeiss 510, 40×/1.3 oil objective) were acquired at each ipsilateral and contralateral region as identified in Figure 1A. For skeleton analysis, the maximum intensity projection of the iba-1 positive channel was enhanced to visualize all microglia processes followed by noise de-speckling to eliminate single-pixel background fluorescence. The resulting image was converted to a binary and then skeletonized using Image J software (Figure 1B). The AnalyzeSkeleton plugin (http://imagejdocu.tudor.lu/) was then applied to all skeletonized images to collect data on the number of endpoints per frame (Figure 1B, blue) and process length (Figure 1B, orange). These data were used as measures of microglia morphology based on previous reports showing reduced microglia process branching complexity and process length in response to injury [14-16]. In addition, others have assessed the microglia process length of single cells using a similar type of analysis [16]. The number of cell somas per frame was used to normalize all process endpoints and process lengthes.

Confocal images were acquired from an additional cohort of slices as described above in ipsilateral and matching contralateral regions. Using Image J, the minimum threshold (0–255) was adjusted for each contralateral image to exclude background fluorescence (average minimum across all images was 18.5 ± 5); thresholding values were constant between matching contralateral and ipsilateral regions. The percent area and mean fluorescence intensity for each threshold image were multiplied to result in the total fluorescence intensity (TFI) for each image. Cells were counted in each image to result in TFI/cell.

### Live cell imaging in acute brain slices

CX_3_CR_1_-GFP ^−/+^ mice underwent the tMCAO procedure as previously described. After 24 h reperfusion, animals were killed, and brain tissue was sliced into 300-μm coronal sections in ice-cold aCSF using a vibratome (Leica VT 1200S, Leica Microsystems, Wetzlar, Germany). The composition of aCSF used for brain removal, brain slicing and live cell imaging was (in mM): KCl 3, NaCl 120, MgCl_2_ 1, NaHCO_3_ 26, NaH_2_PO_4_ 1.25, glucose 10, CaCl_2_ 2 and 400 μM L-ascorbic acid, with osmolarity at 300–305 mOsm, equilibrated with 95% O_2_ and 5% CO_2_ (pH 7.4). Slices were kept at RT in equilibrated aCSF until transferred to the microcopy chamber for live cell imaging. Although it is widely accepted that acute brain slices are viable for ~6 h depending on the animal strain, age and aCSF composition, microglia imaging was limited to a 2-h window after slicing to limit slicing effects as a confounding variable to data collection. The microscope chamber was continuously perfused (2–3 ml/min) with equilibrated aCSF via a peristaltic pump (Miniplus 3, Gilson, Middleton, WI). The chamber temperature was maintained at 35 ± 2°C using a single-line solution heater and DC power supply (SH-28B, Warner Instruments, Hamden, CT; 1735A, BK Precision, Yorba Linda, CA). The necrotic tissue, the result of the tMCAO procedure, was readily apparent by the distinct lack of cells and tissue degradation under bright field and fluorescent illumination. Live microglia images were acquired in the ipsilateral peri-infarct region, just proximal to the necrotic core (region 2), the ipsilateral hemisphere distal to the necrotic core (region 4), and the contralateral hemisphere (region C).

### Sholl analysis

Z-stack confocal live microglia images (15–23 μm) were acquired at 1-μm intervals using a 60× Nikon Objective (NIR Apo 60×/1.0w). Consecutive Z-stack Images were converted to a maximum intensity projection image using Image J software. Using the Image5D plugin (Image J, NIH), z-stack images were condensed into a maximum intensity projection image over which concentric circles were drawn (concentric circles plugin, Image J), centered on the soma, beginning at 5.5-μm radii and increasing 2 μm with every circle. Sholl analysis was manually performed for each cell by counting the number of intersections between microglia branches and each increasing circle to create a Sholl plot. From these data we determined the process maximum (N_m_, the maximum number of intersections for the cell), the critical value (Cr, the distance from the soma where N_m_ occurred), the maximum branch length (μm, the maximum radius at which a branch intersection occurred) and the number of primary branches (N_p,_ the number of branches that originated from the microglia soma). From these parameters, a Shoenen ramification index was calculated (N_m_/N_p_) to quantify cell branching density [24,25]. Additional measures to characterize each cell included the number of branch endpoints (manual count) and cell soma area (Image J Analysis).

### Measuring microglia process movement

To assess microglia activity and process movement, z-stack (20-33-μm) confocal live microglia images were acquired at 3-μm intervals over 8–10 min. The series of z-stack images were compiled into movies using the Image5D plugin (Image J, NIH) and z-stack maximum intensity projection function, resulting in 300–500 images/movie. Movies were acquired in similar regions as described above. Each cell was assessed for process movement and designated as either a cell with a moving or non-moving process. Process activities were visually assessed by counting the number of events (process extension/retraction) occurring within a fixed region of interest (10 × 10 pixels) over time using SparkAn software (custom software created by Dr. Adrian Bonev, University of Vermont).

### Western blot

An independent cohort of animals underwent the tMCAO procedure and periods of reperfusion. Whole brains were removed from euthanized animals at four time-points: ischemia (*n* = 5), ischemia with 8-h (*n* = 4), 24-h (*n* = 5) and 72-h reperfusion (*n* = 3) and sliced into four 2-mm coronal sections (Braintree Scientific, BS-A 5000) beginning at the bregma (3 mm). The middle slices (bregma 1.5 mm to −2.5 mm) were immediately transferred to slides cooled by dry ice. Three ipsilateral and contralateral samples were punched (Harris Micro-punch, Sigma-Aldrich, Z708658) and samples were stored at −80°C. To extract protein, samples were homogenized in ice-cold RIPA buffer containing RIPA lysis buffer (EMD Milipore, 20–188) and 1% each of 1 mmol/l PMSF (Sigma-Aldrich, P7626), 200mM Na_3_VO_4_ (Sigma-Aldrich, S6508) and protease inhibitor cocktail (Sigma-Aldrich, P8340) followed by 2-h digestion at 4°C. Supernatant was collected after 15-min centrifugation and analyzed for protein concentration using the Bradford method; bovine serum albumin was used as standard. Then 50 μg of protein of each sample was separated on a precast gel (mini-protean TGX Any Kd, 15 well, 15 μl/well; BioRad, 4569036) and electrophoretically transferred to a nitrocellulose membrane. Non-specific binding was blocked with 5% non-fat dry milk in Tris-buffered saline solution with Tween 0.1% (TBS-T) for 1 h at room temperature. The membrane was then incubated overnight at 4°C with a solution of primary mouse anti-iNOS antibody (1:100, BD Transduction, 610328) in 5% non-fat milk in TBS-T. Each membrane was washed and then incubated with 1:1,000 donkey anti-mouse horseradish peroxidase secondary antibody (Santa Cruz, SC2314) for 1 h at room temperature. A chemiluminescent assay kit (ChemiGlow, Fisher Scientific, 50921415) was used to detect immunoreactive bands, and the intensity of all bands at 130 Kda was estimated by densitometry analysis using AlphaView SA. All densitometry measurements were normalized to β-actin using antibody (1:30,000, Sigma-Aldrich, A3854). Data are presented as a percent change of ipsilateral iNOS expression from the contralateral hemisphere.

### Statistical analysis

Statistical significance between ipsilateral and contralateral experimental groups was determined by Student’s *t*-test. A one-sample *t*-test was used to assess ipsilateral iNOS expression relative to the contralateral hemisphere. One-way ANOVA with Bonferroni post-hoc testing was used to test for differences among sham, ipsilateral and contralateral or among multiple regions in the ipsilateral hemisphere. A two-way ANOVA was used to assess the spatiotemporal relationship between measures of microglia morphology and cerebral injury after ischemic stroke and periods of reperfusion. All data are presented as the mean ± standard error of mean (SEM). GraphPad Prism 5 was used for all analyses.

## Results

### Quantifying microglia morphology in immunofluorescent images of fixed brain tissue

We assessed microglia morphology during stages of ischemic stroke (ischemia, ischemia with 8 and 24 h of reperfusion) in a striatal region (region 1) and three cortical regions (regions 2–4) as well as matching (mirrored) contralateral regions, as shown in the TTC image representative after ischemia and 24 h of reperfusion (Figure 1A). In this study, region 4 is considered the healthiest region in the ipsilateral hemisphere as it is distal to the developing brain lesion, whereas regions 1, 2 and 3 become encompassed by the peri-infarct region during the temporal evolution of brain infarct. Representative confocal maximum intensity projections, binary, skeletonized (necessary precursors to skeleton analysis) and skeleton analysis images are shown in Figure 1B. Because microglia branching complexity and process length become attenuated (de-ramification) in response to mechanical and chemical injury [14-16], we summarized the number of process endpoints (Figure 1B, blue) and process length (Figure 1B, orange) from the Analyze Skeleton Image J plugin (NIH) data output summary. Composite images of microglia (Iba-1, green), neurons (NeuN, blue) and cell death (propidium iodide, red) are shown in Figure 1C to illustrate the context of typical brain injury in regions 1–4. While propidium iodine staining (red) of cell death is noticeably present in brain regions closest to the ischemic core (regions 1–3) relative to healthier region 4, NeuN staining is progressively decreased; both staining methods are indicators of increased cell death [23,26].

### Microglia cells have a regional pleomorphic response to cerebral ischemia

**Figure 2:**
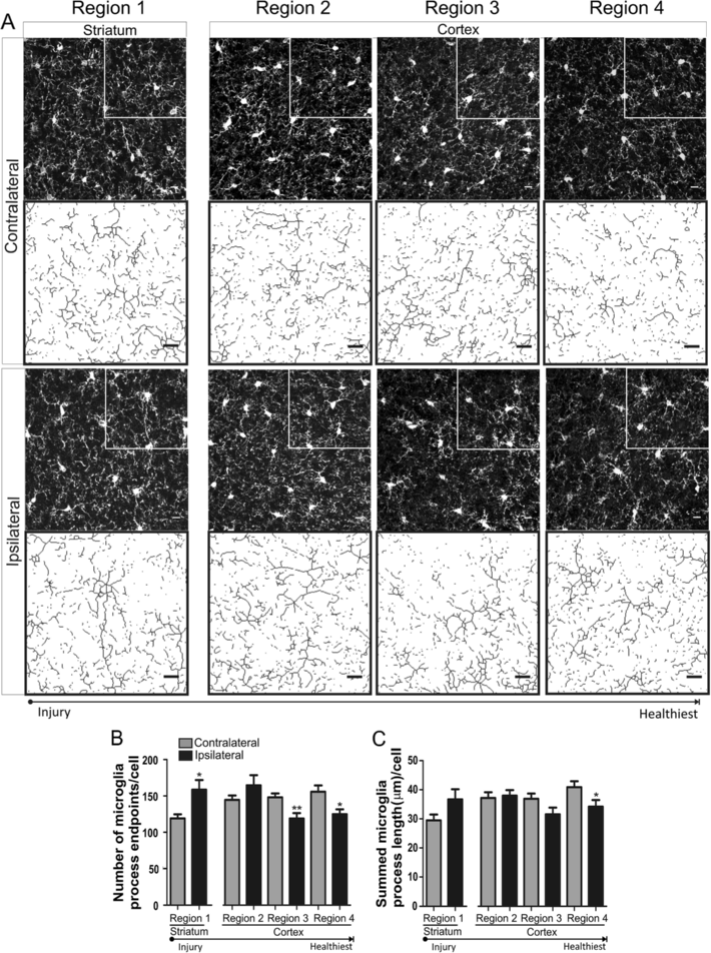
**Microglia are pleomorphic after 60 min of focal ischemia. A** Representative confocal maximum intensity projection images of iba-1 positive microglia and magnified skeletonized inset in brain regions (1 striatum; 2–4 cortex) after 60 min of focal ischemia. **B** Microglia process endpoints/cell are significantly increased in ipsilateral region 1 but decreased in regions 3 and 4 versus matching contralateral regions. **C** Microglia process length/cell was significantly decreased in ipsilateral region 4 versus contralateral hemisphere. Data were tested for statistical differences between ipsilateral vs. contralateral regions using Student’s *t*-test (*n* = 10, *p < 0.05, **p < 0.01 and ***p < 0.001). All data are mean ± SEM. Scale bar = 10 μm.

We quantified microglia morphology in striatal and cortical brain regions (regions 1–4, in Figure 1A) after 60 min of focal ischemia without reperfusion. Representative confocal images of all regions, with enlarged skeletonized quartiles, are shown in Figure 2A. The number of microglia process endpoints/cell was significantly increased (33%) in region 1 when compared to matching contralateral regions (Figure 2B), an indication that microglia branching complexity is increased in the striatal region, a region most affected after focal ischemia induced by the tMCAO procedure. In contrast, the number of endpoints/cell was significantly reduced in the cortex, a 30% reduction in region 3 and 20% in region 4. Microglia process length/cell was not significantly increased in the striatum (*p* = 0.08) but was significantly reduced by 16% in cortical region 4 when compared to the matching contralateral region (Figure 2C). Combined, these data suggest a pleomorphic microglial response—both hyper-ramified and de-ramified—to focal cerebral ischemia in the ipsilateral hemisphere.

### Microglia transition towards a de-ramified morphology after ischemic stroke and 8 h of reperfusion

**Figure 3:**
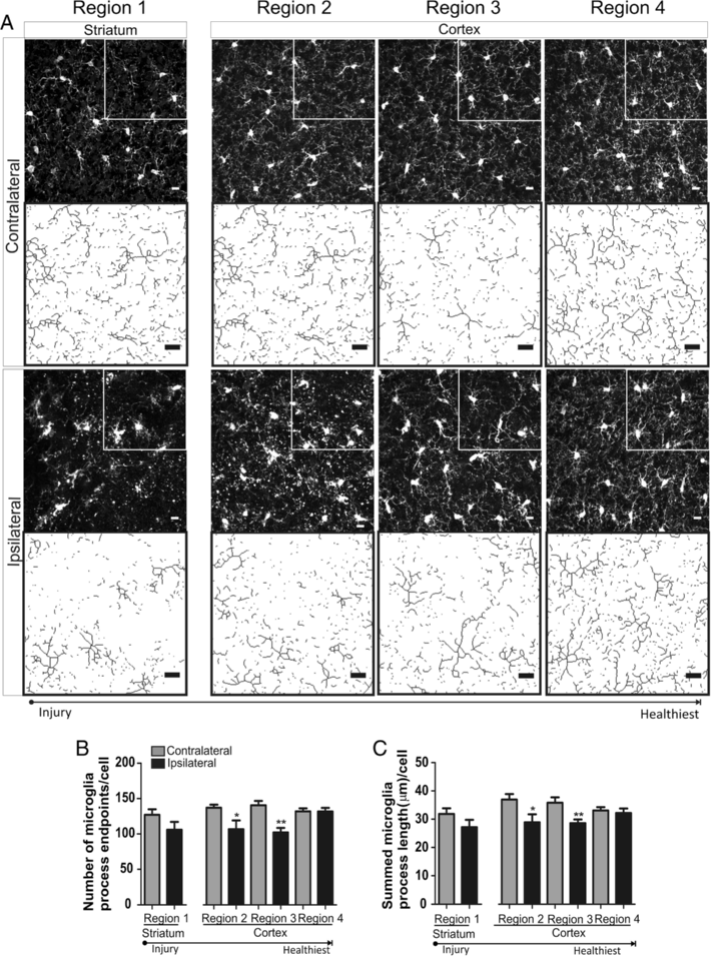
**Microglia are de-ramified in brain regions proximal to cerebral injury after ischemic stroke and 8 h of reperfusion. A** Representative confocal maximum intensity projection images of iba-1 positive microglia and magnified skeletonized inset in brain regions (1 striatum; 2–4 cortex) after 60 min of focal ischemia and 8-h reperfusion. **B** The number of microglia process endpoints/cell was significantly reduced in ipsilateral regions 2 and 3 versus matching contralateral regions. **C** Microglia process length/cell was significantly reduced in ipsilateral regions 2 and 3 versus matching contralateral hemispheres. Data were tested for statistical differences between ipsilateral vs. contralateral regions using Student’s *t*-test (*n* = 7 *p < 0.05 and **p < 0.001). All data are mean ± SEM. Scale bar = 10 μm.

We were also interested in the type (ramification vs. de-ramification) and extent of morphological changes to microglia after ischemic stroke and 8 h of reperfusion. Brain regions were consistent among time points (Figure 1A, regions 1–4), and representative confocal images with skeletonized quartiles are shown in Figure 3A. Microglia process endpoints/cell were significantly decreased after ischemic stroke and 8 h of reperfusion in regions 2 and 3, by 16% and 27%, respectively, when compared to matching contralateral regions (Figure 3B). We observed similar significant decreases in ipsilateral microglia process length/cell in regions 2 and 3 versus matching contralateral regions (22% and 20%, respectively; Figure 3C). Microglia are de-ramified in cortical brain regions after ischemic stroke and 8 h of reperfusion.

### Ipsilateral microglia are de-ramified after ischemic stroke and 24 h of reperfusion

**Figure 4:**
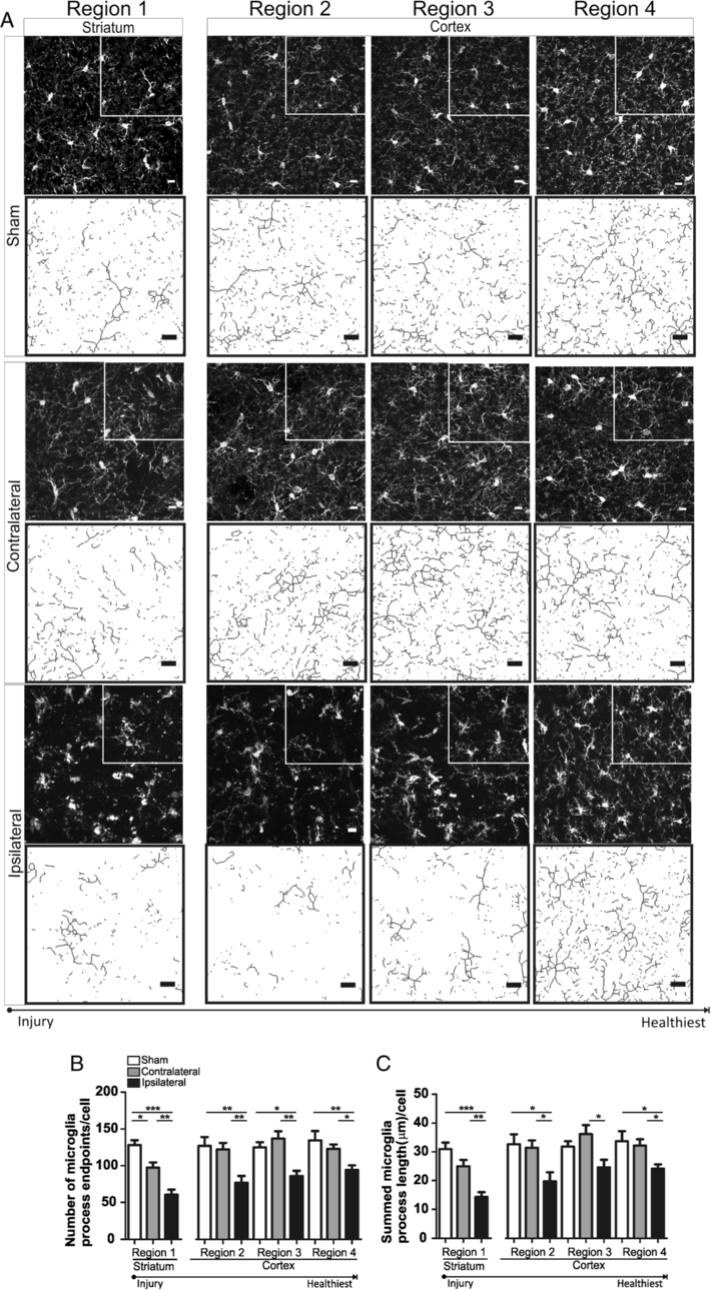
**Microglia ramification is significantly decreased in all ipsilateral regions after ischemic stroke and 24-h reperfusion. A** Representative confocal maximum intensity projection images of iba-1 positive microglia and magnified skeletonized inset in brain regions (1 striatum; 2–4 cortex) after 60 min of focal ischemia and 24-h reperfusion (Figure 1A). **B** Microglia process endpoints/cell are significantly reduced in ipsilateral regions 1–4 versus matching contralateral and sham regions and also significantly reduced in contralateral region 1 versus matching sham. **C** Microglia process length/cell is significantly decreased in all ipsilateral regions (1–4) versus matching contralateral regions. Ipsilateral microglia process length/cell was significantly reduced versus sham in regions 1, 2 and 4, and no significant differences were detected in contralateral regions versus matching sham. *n* = 6/sham group; *n* = 10/contralateral and ipsilateral groups. Data were tested for statistical differences between ipsilateral, contralateral and sham regions using one-way ANOVA and Bonferroni post-hoc testing (*p < 0.05, **p < 0.001, ***p < 0.0001). All data are mean ± SEM. Scale bar = 10 μm.

We assessed microglia morphology after ischemic stroke and 24 h of reperfusion, a time point during acute stroke where, in a mouse model, cerebral injury is considered fully evolved [26]. The number of microglia process endpoints/cell was significantly reduced in all ipsilateral regions compared to their respective contralateral regions, a 34%, 37%, 37% and 24% reduction, respectively, in regions 1, 2, 3 and 4 (Figure 4B). We also show a 25% decrease in the number of microglia process endpoints/cell in the contralateral region when compared to sham. Similarly, microglia process length/cell was significantly reduced in all ipsilateral regions when compared to matching contralateral regions, a 39%, 36%, 33 and 24% reduction in regions 1, 2, 3 and 4, respectively (Figure 4C). However, there were no significant differences noted in the microglia process length/cell between matching contralateral and sham brain regions.

### Microglia morphology is spatially and temporally associated with the evolution of cerebral injury during ischemic stroke and reperfusion

**Figure 5:**
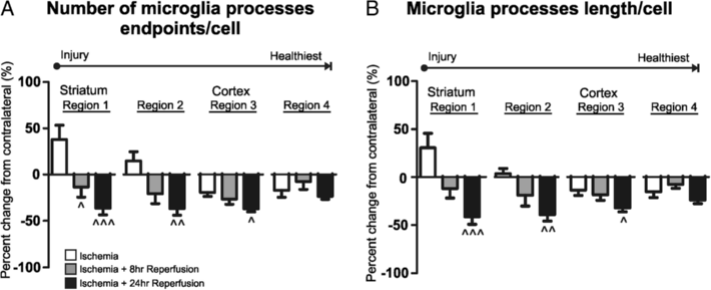
**The spatiotemporal relationship of microglia morphological changes after ischemic stroke and reperfusion.** The percent change in the number of microglia process endpoints/cell (**A**) and microglia process length/cell (**B**) between the contralateral and ipsilateral regions (1–4) was calculated for each time point (ischemia, ischemia and 8 h of reperfusion, and ischemia and 24 h of reperfusion) for two-way ANOVA analysis. **A** There was a significant interaction effect between brain regions and time points (F = 4.17, *p* < 0.001, two-way ANOVA). Main effects, region and time of reperfusion were also significant (F = 3.70, *p* < 0.05 and F = 20.86, *p* < 0.0001, respectively). There were significant changes in microglia process endpoints/cell after ischemia and 8 h of reperfusion in region 1 (^*p* < 0.05 vs. ischemia) and after ischemia and 24 h of reperfusion in region 1, 2 and 3 (**^^^***p* < 0.0001, **^^***p* < 0.001 and ^*p* < 0.05 vs. ischemia). There were no significant changes in microglia process endpoints/cell in any region when comparing the 8-h reperfusion and 24-h reperfusion time points (one-way ANOVA analysis with Bonferroni post-hoc). **B** There was a significant interaction effect between brain regions and time point (F = 2.6, *p* = 0.01, 2-way ANOVA) and a significant main effect for time of reperfusion but not region (F = 21.16, *p* < 0.0001 and F = 1.54, *p* = 0.20, respectively). There were significant changes in microglia process endpoints/cell after ischemia and 24 h of reperfusion in regions 1, 2 and 3 (**^^^**p < 0.0001, **^^***p* < 0.001 and ^*p* < 0.05 vs. ischemia) but no significant changes in any region when comparing after ischemia and 8 h of reperfusion vs. ischemia only or ischemia and 8 h of reperfusion and ischemia and 24 h of reperfusion (one-way ANOVA with Bonferroni post-hoc analysis).

To further elucidate the spatiotemporal relationship between microglia morphological responses and the evolution of cerebral injury after ischemic stroke and reperfusion, we analyzed microglia morphology data as a percent change from contralateral in all brain regions and across all time points. Regarding the number of process endpoints/cell and process length/cell, we identified a significant interaction effect (F = 4.17, *p* < 0.001) and significant main effect for the region and time of reperfusion (F = 3.70, *p* < 0.05 and F = 20.86, *p* < 0.0001, respectively). With further examination, there was a significant percent change in the number of microglia process endpoints/cell between the ischemia and ischemic stroke with 8 h of reperfusion in region 1 (^*p* = 0.05 vs. ischemia) and changes in morphology in regions 1, 2 and 3 after ischemic stroke with 24 h of reperfusion (**^^^**p < 0.0001, **^^***p* < 0.001 and ^*p* < 0.05 vs. ischemia; Figure 5A). Regarding microglia process length/cell, we identified a significant interaction effect (F = 2.6, *p* = 0.01) and a significant main effect for the time of reperfusion but not the region (F = 21.16, *p* < 0.0001 and F = 1.54, *p* = 0.20, respectively). Morphological changes were observed in brain regions 1–3 after ischemia and 24 h of reperfusion (**^^^***p* < 0.0001, **^^***p* < 0.001 and ^*p* < 0.05 vs. ischemia; Figure 5B). Interestingly there were no statistically significant differences in microglia morphology in any brain regions between the later two time points (ischemic stroke and 8 h of reperfusion and ischemic stroke and 24 h of reperfusion) in either measure of microglia morphology (endpoints/cell and process length/cell).

### The morphology of live microglia is altered after ischemic stroke and 24 h of reperfusion

**Figure 6:**
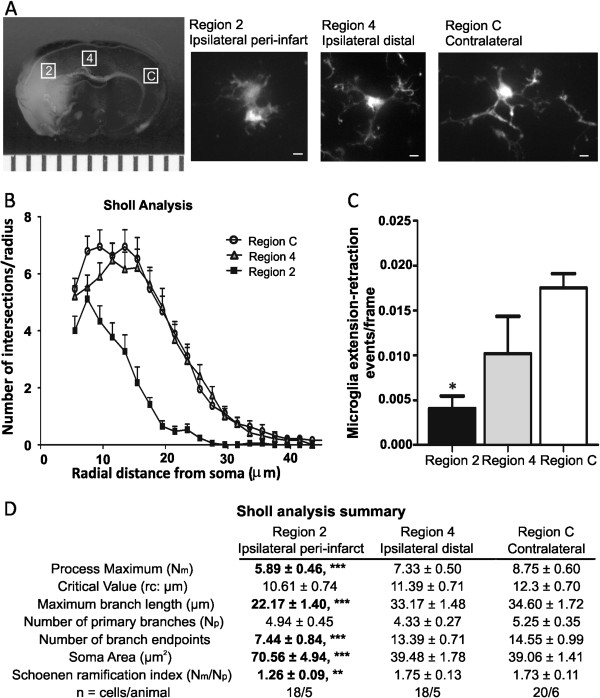
**Microglia ramification and activity are decreased in the peri-infarct region after ischemic stroke and 24-h reperfusion. A** Exemplary maximum projection intensity images of live microglia in brain regions 2 (ipsilateral peri-infarct region), 4 (ipsilateral distal to necrotic core) and C (contralateral region). Brain regions for image acquisition in relation to cerebral injury are as shown in a representative TTC image. **B** Sholl analysis plot of microglia. **C** Microglia process movement is significantly decreased in region 2 (ipsilateral peri-infarct) after ischemic stroke and 24-h reperfusion. **D** Sholl analysis data summary. Microglia nearest to the infarct core have a significantly increased soma size and decreased maximum branch length, number of branch endpoints, process maximum and ramification index when compared to cells in the contralateral hemisphere. Sample size is as listed in the figure. Data were tested for statistical differences between groups, using one-way ANOVA and Bonferroni post-hoc testing (*p < 0.05, **p < 0.001, ***p < 0.0001). All data are mean ± SEM. Scale bar = 5 μm.

We studied live microglia morphology in acute brain slices after ischemic stroke and 24 h of reperfusion. The main purpose of these experiments was to quantify the microglia ramification in live tissue subjected to ischemic stroke with 24 h of reperfusion and, where appropriate, corroborate our previous findings from skeleton analysis of immunohistochemical images. However, unlike immunohistochemical staining of fixed tissue, skeleton analysis in real-time experiments was not possible because of variability in fluorescence intensity throughout the cell (soma vs. processes). Therefore, we analyzed microglia morphology on a cell-by-cell basis using Sholl analysis—a well-established technique to quantitatively analyze neuron and astrocyte processes and branching complexity [24,25]. Images of live microglia were collected from brain regions 2 and 4 (to parallel immunohistochemistry regions 2 and 4) as well as from a single contralateral region C (Figure 6A) after ischemic stroke and 24 h of reperfusion. A Sholl analysis plot (Figure 6B) clearly illustrates that the branching profile of microglia in the region nearest the necrotic tissue (region 2) is shifted to the left, an indication of decreased branching as a function of distance from the soma. Data summarized from the Sholl analysis revealed that microglia in region 2 had significantly fewer process endpoints and significantly reduced maximum branch length (49 and 40%, respectively) when compared to cells located in the contralateral region (Figure 6D). These data are in parallel to those obtained from the skeleton analysis of fixed tissue. However, in contrast to skeleton analysis, there were no significant differences in morphology observed between region 4 and the contralateral hemisphere. Lastly, calculation of the Schoenen ramification index, derived from the Sholl analysis, reveals that microglia are de-ramified by 27% in region 2 when compared to cells in the contralateral region.

### Microglia activity and process movement is blunted in the ipsilateral hemisphere after ischemic stroke and 24 h of reperfusion

In addition to live cell morphology, we examined microglia process activity in the ipsilateral and contralateral hemispheres after ischemic stroke and 24 h of reperfusion. We found that cells imaged in the contralateral hemisphere possessed moving processes (4/4), whereas the number of cells with moving processes was greatly reduced in both ipsilateral regions (5/13 and 3/18 in the distal and peri-infarct regions, respectively). We measured process extension-retraction events in cells identified as actively moving. The number of microglia process extension-retraction events was significantly blunted by 76% in the peri-infarct region when compared to the contralateral hemisphere (Figure 6C).

### Diverse microglia morphologies are not accompanied by altered inducible nitric oxide synthase expression during ischemia and the first 24 h of reperfusion

**Figure 7:**
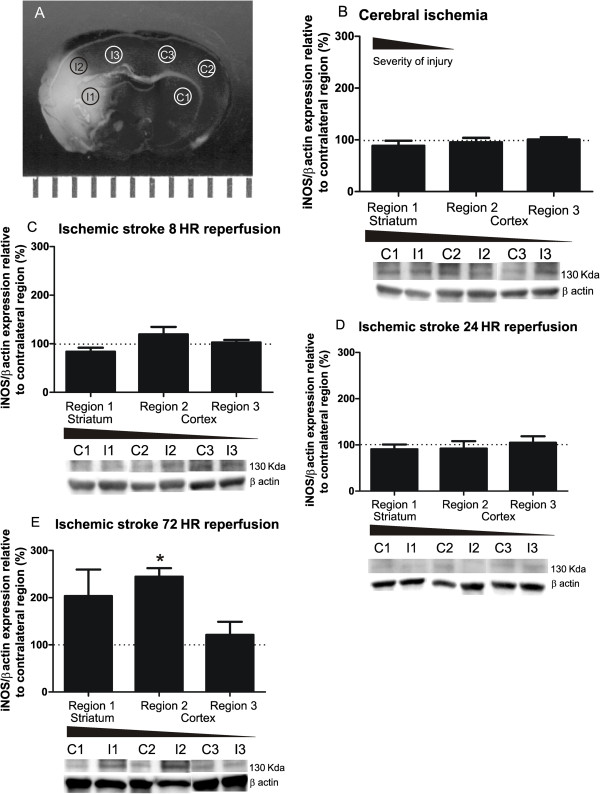
**Tissue iNOS expression is not altered during the first 24 h after cerebral ischemia and reperfusion. A** Brain tissue for Western blot analysis of iNOS expression was acquired in regions 1–3 (1 striatum; 2, 3 cortex) as illustrated in the TTC image of a typical area of injury after ischemic stroke and 24-h reperfusion. Brain regions were conserved across all time points in the ipsilateral and contralateral hemispheres. **B** iNOS expression, normalized to β actin, is not significantly changed relative to the contralateral hemisphere (100%) after 60 min of focal ischemia (*n* = 5), **C** nor after ischemic stroke and 8 h (*n* = 4) or **D** ischemic stroke and 24 h of reperfusion (*n* = 5). **E** iNOS expression was significantly increased in the cortical ipsilateral region proximal to the necrotic core after 60 min of ischemia and 72 h of reperfusion (*n* = 3). Data were tested for statistical differences in iNOS expression from contralateral (100%) using a one-sample t-test (*p < 0.05). All data are mean ± SEM.

To complement to our investigation into microglia morphological responses to ischemic stroke and reperfusion, we investigated microglia proinflammatory function following focal cerebral ischemia and during the first 24 h of reperfusion of ischemic stroke. Although it is well known that NOS imbalance (iNOS/eNOS versus nNOS) following stroke may exacerbate cerebral injury, the microglial contribution toward such an imbalance during ischemia and the first 24 h of reperfusion remains unclear [27,28]. As morphology distinguishes between infiltrating immune cells and microglia as well as subpopulations of responding microglia, we also sought to distinguish the relationship between iNOS expression and regions of altered microglia morphology after ischemic stroke. Tissue was collected in consistent ipsilateral and contralateral brain regions to measure iNOS protein expression at all time points, as shown in Figure 7A. Analysis of Western blots revealed no significant differences in iNOS expression in any ipsilateral region compared to the matching contralateral region in response to cerebral ischemia (Figure 7B) or during the first 8 and 24 h of reperfusion (Figure 7C and D). Data collection was extended to a 72-h reperfusion timepoint where we observed a significant increase in ipsilateral iNOS expression proximal to the necrotic core (Figure 7E). These data suggest that ipsilateral iNOS expression in cells, to include microglia, is not a significant proinflammatory factor during the first 24 h of ischemia and reperfusion and therefore cannot be correlated to diverse microglia morphologies present in concurrent ipsilateral regions.

### Phagocytic marker CD11b is increased in regions of hyper-ramified and de-ramified microglia

**Figure 8:**
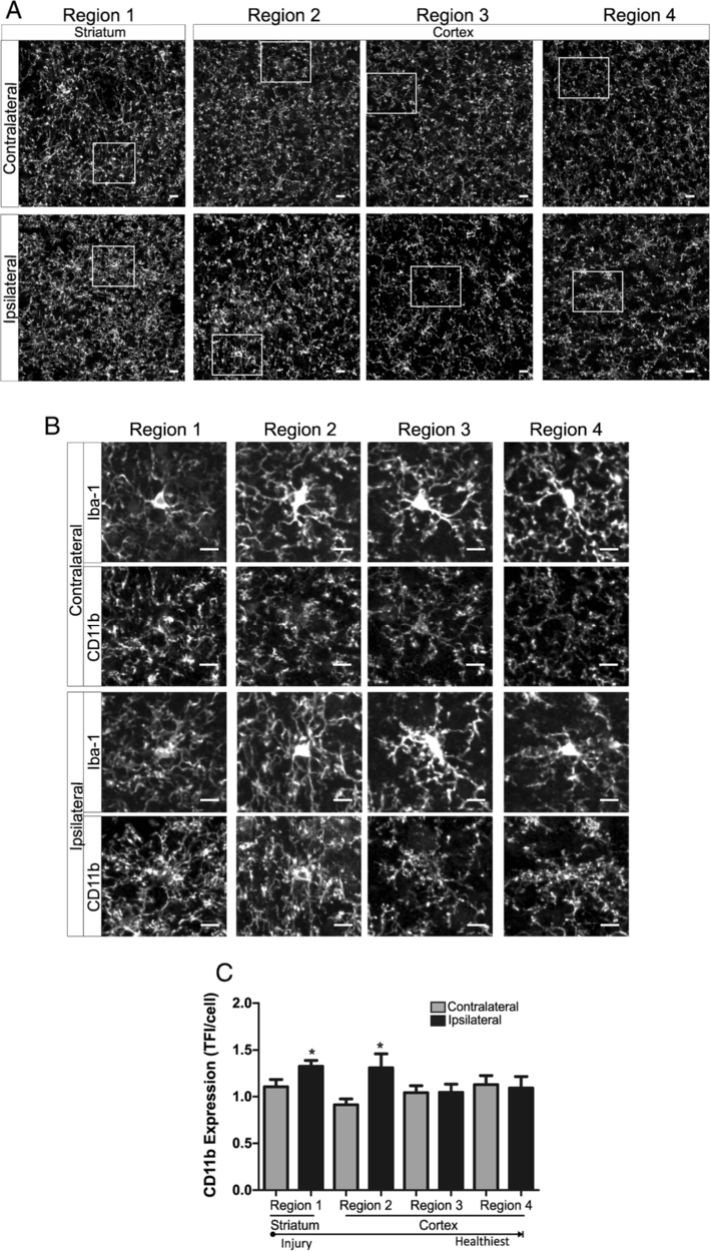
**CD11b expression is increased in regions of hyper-ramified but not de-ramified microglia after 60 min of focal cerebral ischemia. A** Representative confocal maximum intensity projection images of CD11b expression in ipsilateral and contralateral brain regions (1 striatum; 2–4 cortex) after 60 min of focal ischemia. **B** Enlarged image of microglia (top panel, iba-1) and corresponding CD11b expression (bottom panel) from boxed region as depicted in (**A**). **C** Microglia CD11b expression was significantly increased in ipsilateral regions 1 and 2 versus matching contralateral hemispheres. Data were tested for statistical differences between ipsilateral vs. contralateral regions using Student’s *t*-test (*n* = 10, *p < 0.05). All data are mean ± SEM. Scale bar = 10 μm.

**Figure 9:**
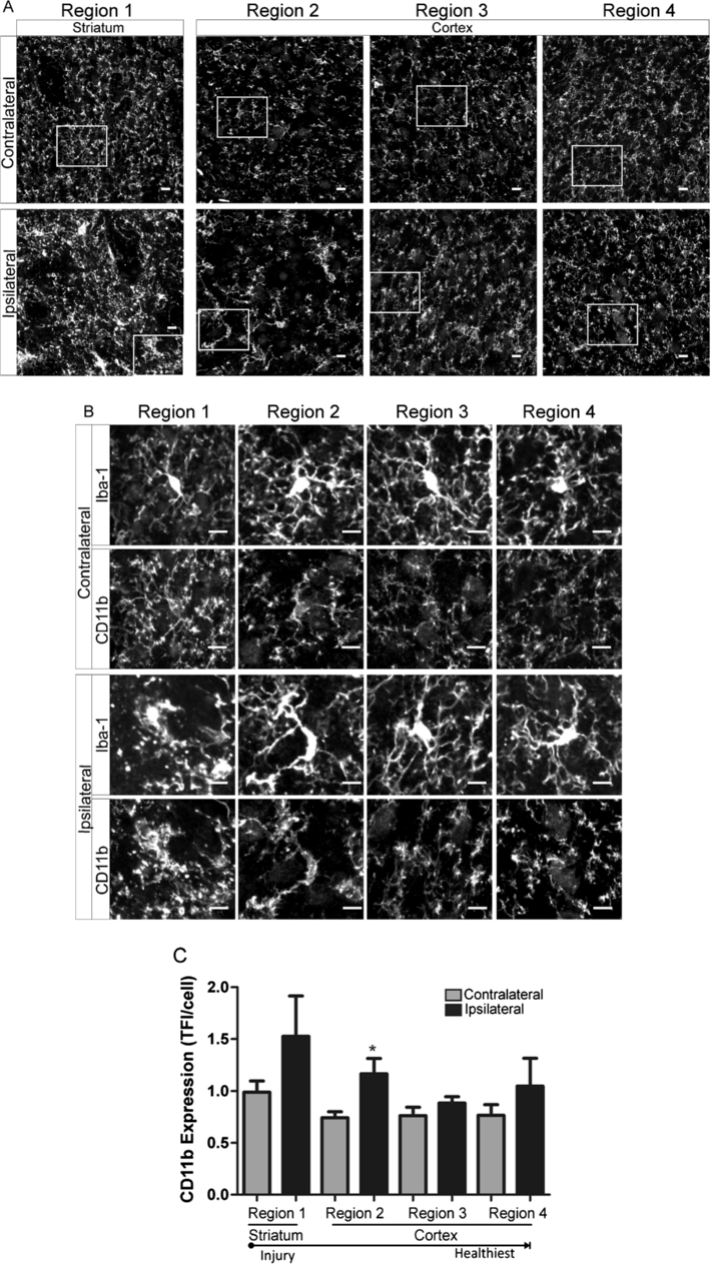
**Microglia CD11b expression is increased after ischemic stroke followed by 8 h of reperfusion in cortical regions proximal to the necrotic core. A** Representative confocal maximum intensity projection images of CD11b expression in ipsilateral and contralateral brain regions (1 striatum; 2–4 cortex) after 60 min of focal ischemia and 8 h of reperfusion. **B** Enlarged image of microglia (top panel, iba-1) and corresponding CD11b expression (bottom panel) from boxed region as depicted in (**A**). **C** Microglia CD11b expression was significantly increased in ipsilateral region 2 versus matching contralateral hemispheres. Data were tested for statistical differences between ipsilateral vs. contralateral regions using Student’s *t*-test (*n* = 5, *p < 0.05). All data are mean ± SEM. Scale bar = 10 μm.

**Figure 10:**
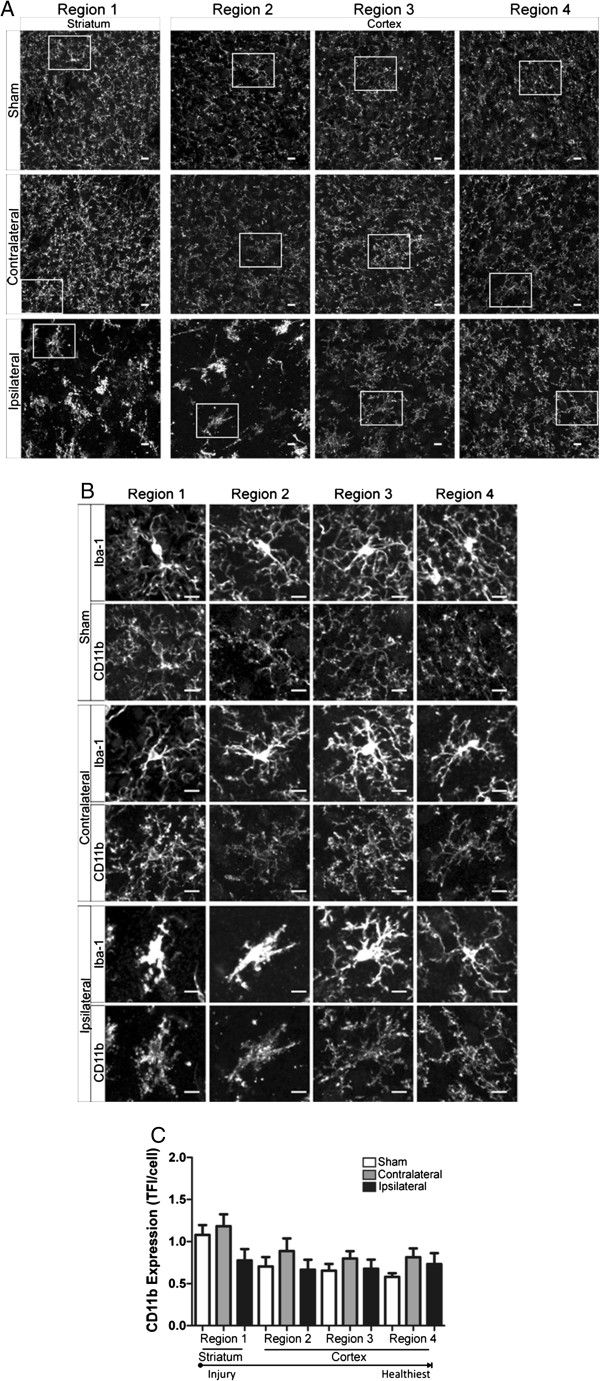
**CD11b expression is not altered in contralateral or ipsilateral regions after ischemic stroke and 24 h of reperfusion. A** Representative confocal maximum intensity projection images of CD11b expression in ipsilateral and contralateral brain regions (1 striatum; 2–4 cortex) after 60 min of focal ischemia and 24 h of reperfusion. **B** Enlarged image of microglia (top panel, iba-1) and corresponding CD11b expression (bottom panel) from boxed region as depicted in (**A**). **C** There were no significant changes in microglia CD11b between any experimental groups (sham, contralateral and ipsilateral) at any region after ischemic stroke and 24 h of reperfusion. Data were tested for statistical differences between ipsilateral vs. contralateral regions using one-way ANOVA analysis (*n* = 5 contralateral/ipsilateral, *n* = 6 sham, *p < 0.05). All data are mean ± SEM. Scale bar = 10 μm.

We studied microglia CD11b expression after focal cerebral ischemia and during the first 24 h of reperfusion in an additional cohort of immunohistochemically prepared tissue samples. Although constitutively expressed by microglia and subsets of granulocytes, increased cell CD11b expression is widely considered a functional response to injury in which CD11b- ligand (e.g., iC3b, fibrinogen) interactions mediate phagocytic functions and generation of cytotoxic proinflammatory mediators [17,29]. Similar to microglia morphology experiments, we measured microglia CD11b expression in striatal and cortical brain regions (regions 1–4, in Figure 1A) at all time points: 60 min of focal ischemia without reperfusion (Figure 8), ischemia and 8 h of reperfusion (Figure 9) and ischemia with 24 h of reperfusion (Figure 10). Representative confocal images of all regions with inset of enlarged microglia iba-1 and CD11b are shown (A) and (B), respectively, in Figures 8, 9 and 10. We illustrate in Figure 8 that hyper-ramified microglia located proximal to the developing necrotic core express significantly more CD11b relative to the matching contralateral region after 60 min of ischemia, a phenomena not observed in more distal regions. In addition, CD11b expression is increased in de-ramified ipsilateral cortical microglia proximal to the necrotic core after ischemic stroke and 8 h or reperfusion (Figure 9). However, after 24 h of reperfusion CD11b expression is not significantly increased in ipsilateral microglia when compared to matching sham or contralateral regions.

## Discussion

Microglia are ramified, dynamic cells in the healthy brain capable of a variety of morphological responses during disease and injury [16,21]. As such, much research is currently centered on elucidating the mediators of altered microglia activity, including both morphology and function, in the healthy brain as well as in models of central nervous system injury [10,11,13,16,21,30,31]. The total progressive morphological and functional changes occurring during ischemia and reperfusion that contribute to cerebral injury after ischemic stroke are poorly understood [2,6,8,32,33]. In the present study, we tested the hypotheses that ischemia and reperfusion would elicit differing microglia morphological responses and that a spatiotemporal relationship exists between microglia morphology and evolving brain injury after ischemic stroke and reperfusion. Quantitative analysis of microglia morphology and associated microglia proinflammatory function revealed two important results: (1) a spatiotemporal relationship exists between microglia morphology and evolving cerebral injury in the ipsilateral hemisphere after ischemic stroke and reperfusion and (2) altered microglia function, as measured by increased phagocytic marker CD11b expression, is observed in regions of hyper- and de-ramified microglia morphologies during the evolution of cerebral injury after ischemic stroke and reperfusion.

Distinguishing between microglia and systemic immune cell (macrophage and neutrophil) responses to injury and disease *in situ* is challenging as these cells share surface and intracellular markers [17,18]. However, microglia’s arborized morphology and dynamic processes behavior allow for the discrimination between these cell populations. In this study, we show that iba-1-positive cells had distinctive arborized features after ischemia alone and after ischemic stroke and 8 h of reperfusion. From this observation we concluded that microglia, as opposed to infiltrating cells, are indeed first responders after ischemic stroke and reperfusion, a notion that is supported by recent research [20,34]. Alternatively, the presence of infiltrating macrophages and neutrophils after ischemic stroke and 24 h of reperfusion cannot be ruled out given robust de-ramification and amoeboid-like morphology of a portion of iba-1 positive cells most proximal to the necrotic core.

Cerebral ischemia and ischemia coupled with reperfusion result in differing pathologic mechanisms, both of which contribute to the evolving brain infarct after stroke [4]. In addition, the heterogeneity and related regional vulnerability of brain tissue to cerebral ischemia and ischemia with reperfusion also differentially contribute to brain injury after stroke [33,35,36]. Therefore, we studied the effects of cerebral ischemia and ensuing reperfusion on ipsilateral microglial morphology during stroke. Using an objective skeleton analysis method to assess morphology, we illustrate that microglia have pleomorphic responses to cerebral ischemia, which includes both hyper- and de-ramification, proximal and distal to the developing necrotic core, respectively. Microglia surveillance is limited to specific micro-domains and, unlike astrocytes, has not been shown to be connected through a broader cell-cell network [10,11,13,37]. Therefore, it is not surprising that during initial and localized injury, microglial responses may be diverse, representative of varied inputs from specific micro-domains. Although not investigated here, altered neuronal signaling during ischemia, toxic ATP and glutamate concentrations in the developing necrotic core, and altered cell receptor expression may influence microglia ramification status [14,16,20]. While cell necrosis is localized to the necrotic core, distal regions are subject to spreading cortical depolarization, increased ATP release through astrocytic networks, altered neuronal signaling and variable tissue oxygenation, suggesting that regional exposure to different environmental cues may influence microglia morphological responses [19,34,35,37].

While reperfusion after ischemic stroke is necessary to reduce ischemic injury, reperfusion also initiates a cascade of deleterious events that contributes to secondary brain injury known as reperfusion injury [8,33]. In light of this, we quantified microglia morphology after 8 and 24 h of reperfusion, which would include the first phase of a biphasic opening of the blood–brain barrier as well as early and fully evolved brain lesions in a murine model [3,26]. In contrast to the pleomorphic responses observed during ischemia, our findings suggest that reperfusion elicited only microglia de-ramification. Although striatal microglia were unchanged after ischemic stroke and 8 h of reperfusion, when compared to matching contralateral controls, process ramification was decreased when considering their initial hyper-ramified status. Microglia became incrementally de-ramified in the cortex, in a spatial relationship to the evolving injury, after 24 h of reperfusion and extended exposure to a deleterious milieu in the ipsilateral hemisphere. We expected a more widespread microglial response in the contralateral hemisphere in light of increased blood–brain barrier permeability [3,38] and reports of microglia activation and phagocytic activity in the contralateral hemisphere following ischemic stroke [39,40]. However, we showed that only contralateral striatal microglia but not cortical microglia were de-ramified when compared to comparable sham regions after ischemic stroke and 24 h of reperfusion. Although de-ramified in all ipsilateral regions when compared to their contralateral counterparts, microglia morphology remains diverse within the ipsilateral hemisphere during the first 24 h after reperfusion, an important observation as microglial function may be defined by varied degrees of damage after ischemic stroke [18,41,42]. Future studies addressing microglia morphology in response to their microenvironment are needed.

We conducted additional experiments in live cortical brain slices to further explore the spatial relationship between dynamic microglia morphology and cerebral injury after ischemic stroke and 24 h of reperfusion. Consistent with the fixed tissue data, microglia cells in an *ex vivo* slice preparation were de-ramified in relation to the necrotic core at the 24-h reperfusion time point. The incidence of active microglia was reduced in the ipsilateral hemisphere, and within the cohort of active cells, microglia process activity (protrusion/retraction) in the peri-infarct region was severely blunted when compared to the contralateral hemisphere. In agreement with previous studies [14,34], we suggest that the microglia ramification status is an indicator of microglia process activity after ischemic stroke and reperfusion. Image acquisition of live microglia at 60× magnification was necessary to achieve sufficient resolution to detect microglia processes in the heterozygous CX_3_CR_1_-GFP mouse. We used the Sholl analysis methodology rather than the skeleton analysis approach for multiple reasons. Sholl analysis is ideally suited to the individual analysis of cell morphology rather than batch cell processing used in the skeleton analysis approach. In addition, the conversion of maximum intensity projection images to a binary and then skeletonized image resulted in inaccurate skeletonized structures, likely because of the bright soma of these cells and potential light scattering. Additional limitations were the slicing effects on microglia morphology [15] and bias of the experimenter in individual cell analysis. These limitations were addressed by acquiring live images within a 2-h window following brain slicing and acquiring images in deeper slice layers where live neurons and astrocytes are typically preserved and microglia are less affected by slicing procedure [15]. While the tissue and cell morphology was distinct in region 2/peri-infarct, Sholl analysis of all other microglia cells was performed without knowledge of the brain region, decreasing bias. To our knowledge, this is the first study that applies the Sholl analysis to quantify microglia morphology. Nevertheless, we believe this to be an ideal analysis methodology for the live cells as the results yielded comparable data to that of the skeleton analysis.

In light of the novel identification of both hyper-ramified and de-ramified microglia subpopulations ipsilateral to cerebral injury after stroke, we endeavored to elucidate the role of microglia responses after ischemic stroke and reperfusion by studying associations between microglia form and function. Our investigation included iNOS and CD11b expression as markers of proinflammatory microglia function. Although therapeutic in the healthy brain, excessive NO production (mediated by iNOS) and phagocytosis (mediated by CD11b expression) contribute to secondary injury after ischemic stroke and reperfusion [2,27,43]. Multiple cell types, including microglia, contribute toward cytotoxic concentrations of NO via increased iNOS expression after ischemic stroke [27]; however, the presence of iNOS in ipsilateral brain tissue was not significantly increased until at least 72 h of reperfusion. The fact that we show distinct and diverse microglia morphologies that span the first 24-h period of ischemic stroke is suggestive that iNOS is not a suitable marker of early microglia functional response.

On the other hand, CD11b expression, a well-established marker of increased phagocytic and proinflammatory function [22,29], is an excellent marker of early changes in microglia functional after cerebral ischemia and during early reperfusion. That microglia CD11b expression was significantly increased in regions of both hyper-ramified and de-ramified microglia further supports the notion that both hyper-ramified and de-ramified microglia should be considered when stratifying the microglial “activation” status during disease pathology. However, increased microglia CD11b expression was not exclusively associated with one type of microglia morphology. Thus, microglia phagocytosis may not be a general event but instead, influenced by differing environmental cues within microglia domains, is multi-purposed during spatiotemporal responses to ischemic stroke and reperfusion. For example, targeted phagocytosis directed by ramified microglia processes during cerebral ischemia may influence early synaptic remodeling and containment of developing cerebral injury [20], whereas de-ramified microglia, present after extensive and prolonged injury, contribute toward the clearing of diffuse cellular debris [9].

## Conclusions

Previously, our understanding of microglia morphological responses to ischemia and reperfusion was limited to categorical and anecdotal characterization of microglia morphology [18,41,44-47]. However, it is not surprising that detailed quantitative analyses reveal a diverse microglial response to the complex and deleterious events that encompass ischemic stroke. In this study, we quantitatively characterize the spatial and temporal changes of microglia morphology showing initial increases in microglia ramification followed by progressive microglia de-ramification in ipsilateral brain regions after ischemic stroke and reperfusion. In addition, we demonstrate that such analysis is possible using readily available imaging software. The identification and stratification of microglial morphologic responses, including hyper-ramified morphologies, may be a key element in understanding the full dimension of microglial functional responses in physiologic and pathologic conditions. Future studies that include quantitative analyses of microglial morphology would assist in the identification and stratification of their complex and dichotomous contribution to cerebral injury and recovery during ischemic stroke and reperfusion.

## Competing interests

The authors declare that they have no competing interests.

## Authors’ contributions

HWM contributed to the experimental design, execution of data collection, data analysis, data interpretation, and manuscript writing. JAF contributed to the conception and design of experiments, overall interpretation of data and manuscript writing. All authors read and approved the final manuscript.
